# Driving Forces of the Bubble-Driven Tubular Micromotor Based on the Full Life-Cycle of the Bubble

**DOI:** 10.3390/mi10060415

**Published:** 2019-06-21

**Authors:** Yongshui Lin, Xinge Geng, Qingjia Chi, Chunli Wang, Zhen Wang

**Affiliations:** 1 Hubei Key Laboratory of Theory and Application of Advanced Materials Mechanics, Department of Mechanics and Engineering Structure, Wuhan University of Technology, Wuhan 430070, China; peakspylin@163.com (Y.L.); gengxinge@whut.edu.cn (X.G.); qingjia@whut.edu.cn (Q.C.); 2 “111” Project Laboratory of Biomechanics and Tissue Repair, Bioengineering College, Chongqing University, Chongqing 400044, China; 20121901015@cqu.edu.cn

**Keywords:** micromotor, driving force, hydyodynamics, bubble

## Abstract

Micromotors show many advantages in practical applications, including small size, large push-to-weight ratio, and low power consumption. Micromotors have been widely used in a variety of applications, including cell manipulation, payload delivery, and removal of toxic components. Among them, bubble-driven micromotors have received great attention due to their large driving force and high speed. The driving force of the bubble-driven micromotor movement comes from the four stages of the life cycle of the bubble: nucleation, growth, slip, and ejection. At present, investigators are still unclear about the driving mechanism of the bubble-driven micromotors, the source of the driving force being still especially controversial. In response to this problem, this paper combines the mass transfer model, hydrodynamic theory, and numerical simulation to explain the driving force generated by the various stages of the life-cycle of the bubble. A mass transfer model was used to calculate the driving force of the motor contributed by the bubble nucleation and slip stage. Based on equilibrium of force and conservation of energy, a theoretical model of the driving force of the tubular micromotor in the growth and ejection stage of the bubble was established. The results show that the driving force contributed by the bubble in the nucleation and the slip stage is rather small. However, the stage of bubble growth and ejection provide most of the driving force. On further evaluating the effect of the bubble driving force on the motor speed, it was found that the growth stage plays a major role in the motion of the bubble-driven micromotor. The micromotor velocity based on the driving forces of the full life-cycle of bubbles agrees well with the experimental results.

## 1. Introduction

Micromotors are micro-scale structures that exhibit many advantages in practical applications, including small size, large thrust-to-weight ratio, and low power consumption [1]. Micromotors are widely used in many fields, including cell manipulation [2,3], payload transport [4,5,6], and removal of pollutants [7,8]. Bubble-driven micromotors, which can be divided into Janus particles and tubular structures, have received great attention due to their large driving force and high speed [9]. Tubular micromotors perform typically better than Janus micromotors in locomotion. Moreover, the bubble-driven micromotor does not need to be driven by an external force, has a simple structure, and the propulsion of the micromotor is hardly affected by the ion concentration. The bubble-driven micromotor can be driven to move at a fast speed of more than 100 μm/s [10,11]. For example, a micromotor with zinc as the inner wall and polyaniline as the outer wall can reach a speed of 1000 μm/s. A similar speed can be achieved with a micromotor with Pt/Co/Ti as the inner wall [12].

The bubbles are generated by the reaction between the inner wall of the micromotor and the solution. Among them, hydrogen peroxide is the most commonly used fuel. The driving force of the bubble-driven micromotors comes from the entire life cycle of the bubbles, from the generation to the detachment [9,13]. The life cycle of bubble dynamics includes four stages, namely the nucleation, growth, slip, and ejection within the motor [14,15]. First under the action of a catalytic metal, hydrogen peroxide decomposes to generate gas and form bubbles. The bubbles nucleate at the inner surface of the micromotor wall to form spherical bubbles. Second, after the nucleation of the bubble, the outer boundary of the bubble moves outward under the action of the internal pressure of the bubble. Then the radius of the bubble becomes larger, and the bubble enters the growth stage [16]. Third, the bubble grows to a certain stage, moves to the opening under the action of the fluid, and enters the slip stage. Finally, the bubble moves to the opening of the motor and is ejected away from the tubular motor under different forces.

Investigators are still deep in controversy about the source of the driving force of bubble-driven tubular micromotors. Some authors believe that ejection contributes the most to micromotor motion, while other researchers believe that bubble growth plays a major role. These studies have generally focused on the effects of bubbles on the micromotor motion in a single stage. Solovev et al. [17] suggested that the driving force mainly from bubble ejection accelerates the tubular micromotor to 2 mm/s. Li et al. [12] considered that the driving force of the bubble-driven motor mainly comes from the force at the bubble growth stage. Manjare et al. [18] also reported that bubble growth mainly generated the driving force. Subsequently, this group gave the opposite conclusion. They found that the driving force of bubble growth is much smaller than that of bubble ejection [19]. Fomin’s group [20] proposed that both bubble growth and ejection produced driving forces and established the corresponding theoretical models for prediction. The experimental results showed that the theoretical prediction velocity is two orders of magnitude smaller than the measured velocity. That is, the model underestimates the driving force generated by the bubble. Gibbs et al. [21] suggested that the growth of bubbles provides a driving force for the Janus micromotor.

As for the debate about the source of the driving force of bubble-driven micromotors, it is very important to quantify the driving force generated at each stage of the life cycle of the bubbles. Our previous work established a model of micromotor motion [22] based on geometric asymmetry and fluid viscosity, and predicted the resistance of the cone micromotor immersed in the flow field based on the hydrodynamic model [23]. We also discussed strategies to make fast, efficient bubble-driven micromotors [24], and micromotors powered by biocompatible fuel [25]. The paper further proposed a model of driving force based on the tubular micromotor of the full life cycle of the bubble. In the bubble nucleation stage, the mass transfer model was used to propose the driving force model of the tubular micromotor in the nucleation stage. For the bubble growth stage, the force on the bubble changes as the bubble radius changes. Based on this, a driving force model of the bubble growth stage was proposed. In the slip stage of the bubble, the mass transfer model was used to establish the driving force of the motor. At the ejection stage, a driving force model of the tubular micromotor in the stage was established based on the force. In this paper, the driving force models of bubble nucleation, growth, slip, and ejection are established respectively, and the influence of bubble driving force on the motor motion is further evaluated.

## 2. Materials and Methods 

### 2.1. Bubble Nucleation

According to the classical theory of bubble nucleation [26], the free energy required for the nucleation of bubbles on the inner wall of the micromotor is

(1) $$W=\sigma_{LG}A_{LG}+(\sigma_{SG}-\sigma_{SL})A_{SG}+\Delta G_{V}V_{G}+x(\mu_{G}-\mu_{L})$$

**Figure 1:**
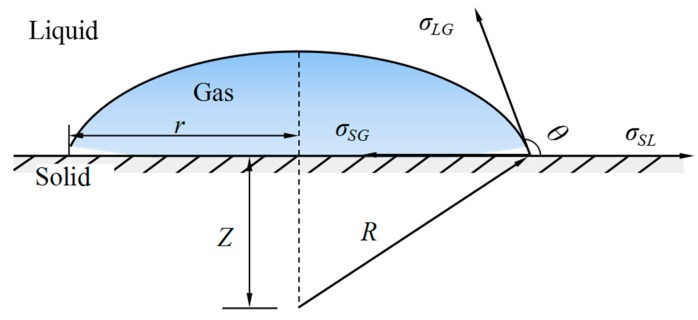
Gas-liquid–solid interface and contact angle of the bubble.

The subscripts *S*, *L*, and *G* represent the solid state, liquid state, and gaseous three-stage state, respectively, where *σ_LG_* is the surface tension coefficient *σ* of the hydrogen peroxide solution required in the calculation. The equilibrium state is shown in Figure 1. According to Young’s equation of the contact angle

(2) $$\sigma_{SL}=\sigma_{SG}+\sigma_{LG}\cos(\pi -\theta )$$

and

(3) $$m=\frac{Z}{R}=\cos(\pi -\theta )=-\cos\theta =\frac{\sigma_{SL}-\sigma_{SG}}{\sigma_{LG}}$$

The liquid and gas contact area *A_LG_* and the solid and gas contact area *A_SG_* are respectively

(4) $$A_{LG}=2\pi R(R-Z)=2\pi R^{2}(1-m)$$

(5) $$A_{SG}=\pi r^{2}=\pi R^{2}(1-m^{2})$$

Gas volume is

(6) $$V_{G}=\frac{1}{3}\pi (3R-H)H^{2}=\frac{1}{3}\pi R^{3}(2-3m+m^{3})=\frac{4}{3}\pi R^{3}F$$

The change of free energy per unit volume can be obtained according to the Laplace equation,

(7) $$\Delta G_{V}=P_{L}-P_{G}=\frac{2\sigma }{R}$$

The difference in chemical potential is

(8) $$\mu_{G}-\mu_{L}=KT\ln\frac{P_{G}}{P_{V}}=KT\ln\frac{P_{L}+2\sigma /R}{P_{L}+2\sigma /R_{C}}$$

The ideal gas equation *PV* = *xKT* can be applied to obtain the free energy required for bubble nucleation

(9) $$W=\frac{4}{3}\pi R^{2}\sigma F+\frac{4}{3}\pi R^{3}\sigma F(P_{L}+\frac{2\sigma }{R})\cdot \ln\frac{P_{L}+2\sigma /R}{P_{L}+2\sigma /R_{C}}$$

The critical radius *R*_c_ can be obtained by the following formula

(10) $$R_{c}=\frac{2\sigma }{\Delta G_{V}}=\frac{2\sigma }{P_{\infty }-P_{G}}$$

*P*_∞_ is the standard atmospheric pressure, and *P*_G_ is the internal pressure of the nucleation bubble.

**Figure 2:**
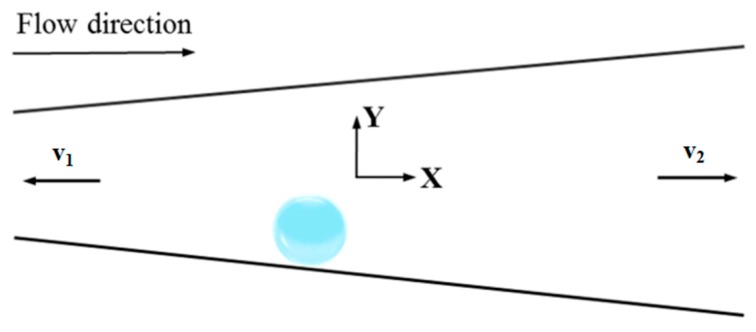
A schematic diagram of the bubble in the nucleation stage.

As shown in Figure 2, the volume of the bubble is partially *V_c_* = 4*π*$R_{c}^{3}$/3. Assuming that the volume of fluid discharged along the left orifice is *fV*_c_, the volume of fluid discharged along the right orifice is (1 − *f*)*V*_c_. As can be seen from the figure, due to the incompressibility of the fluid, the volume *V*_c_ of the nucleation bubble is not the volume of the fluid in the tube. The fluid discharged from the tube flows out from the two openings of the tube, respectively, wherein the ratio of the volume of the left outflow fluid to the total flow is *f*. The parameter f is obtained from FLUENT 18.0 (ANSYS).

The fluid flow rates at the left and right openings are

(11) $$v_{1}=\frac{d(fV_{c})}{dt},\;v_{2}=\frac{d[(1-f)V_{c}]}{dt}$$

In the nucleation stage, the driving force of the tubular micromotor is

(12) $$F_{\mathrm{jet}1}=\frac{d(mv)}{dt}=\frac{d}{dt}(m_{1}v_{1}-m_{2}v_{2})=\frac{d}{dt}[\rho_{l}\cdot fV_{c}\cdot \frac{fV_{c}}{A_{1}}-\rho_{l}\cdot (1-f)V_{c}\cdot \frac{(1-f)V_{c}}{A_{2}}]$$

Substituting into the tubular micromotor two-port area *A*_1_, *A*_2_, and the function of bubble and oxygen production frequency *q*

(13) $$A_{1}=\pi R_{\min}^{2}=\pi {\left(R_{\max}-L\tan\delta \right)}^{2},\;A_{2}=\pi R_{\max}^{2},\;R_{b}={\left(\frac{3qt}{4\pi }\right)}^{\frac{1}{3}}\in [0,R_{c}]$$

Therefore, the driving force in the stage is

(14) $$F_{\mathrm{jet}1}=\frac{\rho_{l}\pi C_{H_{2}O_{2}}^{2}n^{2}L^{2}}{\cos^{2}\delta }{\left(2R_{\max}-L\tan\delta \right)}^{2}[\frac{f^{2}}{\pi {\left(R_{\max}-L\tan\delta \right)}^{2}}-\frac{{\left(1-f\right)}^{2}}{\pi R_{\max}^{2}}]$$

where C_H2O2_ is the concentration of hydrogen peroxide and *f* is a function of time *t*.

### 2.2. Bubble Growth 

**Figure 3:**
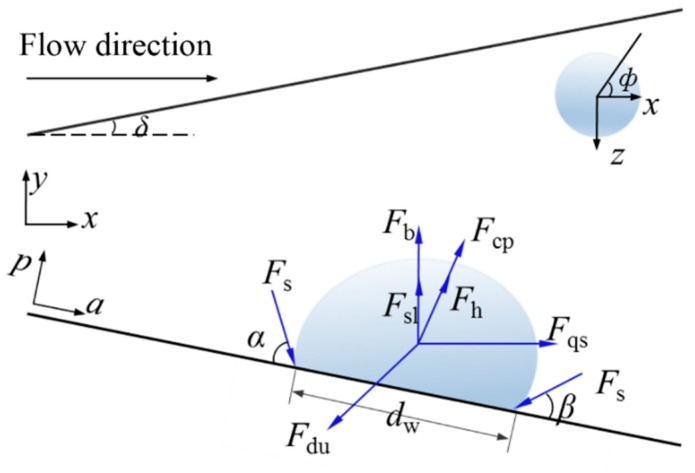
Various forces during the growth stage of the bubble.

After the bubbles nucleate on the tube wall, the generated gas continues to fill into the bubbles and the bubbles gradually grow, as shown in Figure 3.

The force of a bubble along the pipe wall (a axis), perpendicular to the pipe wall (p axis) and parallel to the flow direction (x axis), perpendicular to the flow direction (y axis) is as follows

(15) $$\{\begin{array}{l} \Sigma F_{a}=F_{sa}+F_{qs}\cos\delta +F_{dua}-(F_{b}+F_{sl})\sin\delta \\ \Sigma F_{p}=F_{sp}+F_{qs}\sin\delta +F_{dup}+(F_{b}+F_{sl})\cos\delta +F_{h}+F_{cp} \end{array}$$

(16) $$\{\begin{array}{l} F_{x}=\Sigma F_{a}\cos\delta +\Sigma F_{p}\sin\delta \\ F_{y}=\Sigma F_{p}\cos\delta -\Sigma F_{a}\sin\delta \end{array}$$

*F*_s_ is the surface tension, *F_qs_* is the fluid resistance of the bubble, *F_du_* is the unstable force of the asymmetric growth of the bubble, *F_b_* is the buoyancy, *F_sl_* is the shearing force, *F_h_* is the force caused by the dynamic pressure, and *F_cp_* is the contact pressure. The subscript *a*, *p* denotes a semi-cone angle of a conical micromotor along the wall of the tube and perpendicular to the wall of the tube.

Define contact angle function *γ*(*φ*)

(17) $$r(\phi )=\beta +(\alpha -\beta )\frac{\phi }{\pi },0\leq \phi \leq \pi$$

At the same time, the contact angle function needs to meet the conditions *γ*(0) = *β*, *γ*(π) = *α*, *γ′*(0) = *γ′*(π) = 0. Substituting the contact angle function, the projection of the surface tension on the coordinate axis

(18) $$\{\begin{array}{l} F_{sa}=-\int_{0}^{\pi }d_{w}\sigma \cos\gamma \cos\phi d\phi =-d_{w}\sigma \frac{\pi (\alpha -\beta )}{\pi^{2}-{\left(\alpha -\beta \right)}^{2}}(\sin\alpha +\sin\beta )\\ F_{sp}=-\int_{0}^{\pi }d_{w}\sigma \sin\gamma d\phi =-d_{w}\sigma \frac{\pi }{\alpha -\beta }(\cos\beta -\cos\alpha ) \end{array}$$

At the beginning of the bubble growth, the bubble is spherical. Then *d*_w_ = 2*R*sin*θ*. The volume of the bubble is expressed as $V=1/3\pi R^{3}(2+3\cos\theta -\cos^{3}\theta )$.

While the growth of the bubble is in accordance with *V* = *qt* [27,28], and the bubble radius is equal to

(19) $$R=\gamma t^{n}={\left[\frac{3q}{\pi (2+3\cos\theta -\cos^{3}\theta )}\right]}^{1/3}t^{1/3}$$

The contact angle *θ* = 30° and the semi-cone angle *φ* = 4.5° are applied [29].

The fluid resistance of the bubble is [30]

(20) $$F_{qs}=(\frac{2}{3}+\frac{1}{12}Re)\cdot 6\pi \mu V_{y}R,\;Re=\frac{\rho_{l}V_{y}2R}{\mu }$$

where *Re* is the fluid Reynolds number, *ρ_l_* is the fluid density, *μ* is the fluid viscosity, *R* is the bubble radius, and *V_y_* is the fluid velocity at the bubble center point.

Substituting the average diameter to obtain the axial velocity distribution inside the conical tube

(21) $$V_{y}=\frac{8Q}{\pi h^{2}}-\frac{32Q}{\pi h^{4}}y^{2}=\frac{8Q}{\pi {\left(2R_{\max}-L\tan\delta \right)}^{2}}-\frac{32Q}{\pi {\left(2R_{\max}-L\tan\delta \right)}^{4}}y^{2}$$

Substituting *V_y_* into the projection of surface tension on the coordinate axis, and considering the fluid resistance of small bubbles is 2/3 of the same size solid particles [31], the fluid resistance of the bubble is expressed as

(22) $${F^{\prime }}_{qs}=\frac{2}{3}\cdot 6\pi \mu R[\frac{8Q}{\pi {\left(2R_{\max}-L\tan\delta \right)}^{2}}-\frac{32Q}{\pi {\left(2R_{\max}-L\tan\delta \right)}^{4}}{\left(R\cos\theta -\frac{h}{2}\right)}^{2}]$$

During the growth of the bubble, the deformation of the bubble results in an asymmetrical growth force which can be obtained through a series of simplifications

(23) $$F_{du}=\int_{\Gamma }\rho_{l}(R\ddot{R}+\frac{3}{2}{\dot{R}}^{2})dA=\pi R^{2}\rho_{l}(R\ddot{R}+\frac{3}{2}{\dot{R}}^{2})=-\frac{1}{18}\pi \rho_{l}{\left(\frac{3q}{\pi (2+3\cos\theta -\cos^{3}\theta )}\right)}^{4/3}t^{-2/3}$$

Bubble buoyancy

(24) $$F_{b}=V(\rho_{l}-\rho_{b})g=\frac{1}{3}\pi R^{3}(2+3\cos\theta -\cos^{3}\theta )(\rho_{l}-\rho_{b})g$$

where *ρ_b_* is the density of the gas within the bubble, namely the oxygen density.

The fluid dynamic pressure *F_h_* mainly acts on the circular surface where the bubble contacts the tube wall [32], namely

(25) $$F_{h}=\frac{9}{8}\rho_{l}{V_{y}}^{2}\frac{\pi {d_{w}}^{2}}{4}=\frac{9}{8}\rho_{l}\frac{\pi {d_{w}}^{2}}{4}{\left[\frac{8Q}{\pi {\left(2R_{\max}-L\tan\delta \right)}^{2}}-\frac{32Q}{\pi {\left(2R_{\max}-L\tan\delta \right)}^{4}}{\left(R\cos\theta -\frac{h}{2}\right)}^{2}\right]}^{2}$$

The contact pressure is

(26) $$F_{cp}=\frac{\pi {d_{w}}^{2}}{4}\cdot \frac{2\sigma }{r_{r}}=\frac{\pi {d_{w}}^{2}}{4}\cdot \frac{2\sigma }{5R}$$

The shear lift force is [33]

(27) $$F_{sl}=\frac{2}{3}\cdot 81.2\mu V_{y}R^{2}\kappa^{\frac{1}{2}}/\nu^{\frac{1}{2}}$$

where *μ* is the dynamic viscosity, kinematic viscosity is *ν* = *μ*/*ρ_l_*, velocity gradient $\kappa =\left|\frac{dV_{y}}{dy}\right|=\left|-\frac{64Q}{\pi {\left(2R_{\max}-L\tan\delta \right)}^{4}}y\right|$.

A conversion factor λ needs to be set as the force of the bubble acting on the tubular micromotor. Wang et al. [34] used experimental and molecular dynamics simulations to find that the chemical energy conversion efficiency of bubble-driven motors is about 10^−10^, but the conversion efficiency of bubbles was not studied. Fomin et al. [20] then studied the bubble-driven tubular micromotor and found that the conversion force of the bubble is converted to a tubular motor driving force with a conversion coefficient of about 1/30. The driving force of the tubular micromotor in the stage of bubble growth is

(28) $$F_{\mathrm{jet}2}=\lambda F_{x}$$

### 2.3. Bubble Slip

When the bubble grows up to the moment of contact with the tube wall, the relationship between the bubble radius *R_i_* and the inner diameter *H_i_* of the tube wall at the nucleation point is $H_{i}=2R_{i}\cos\delta$, or $H_{i}=2X_{i}\tan\delta +2R_{\min}$.

If the bubble is kept in the micromotor, there exists the expression *X_i_* − *C*/2 ≥0, *X_i_* + *C*/2 ≤*L*.

**Figure 4:**
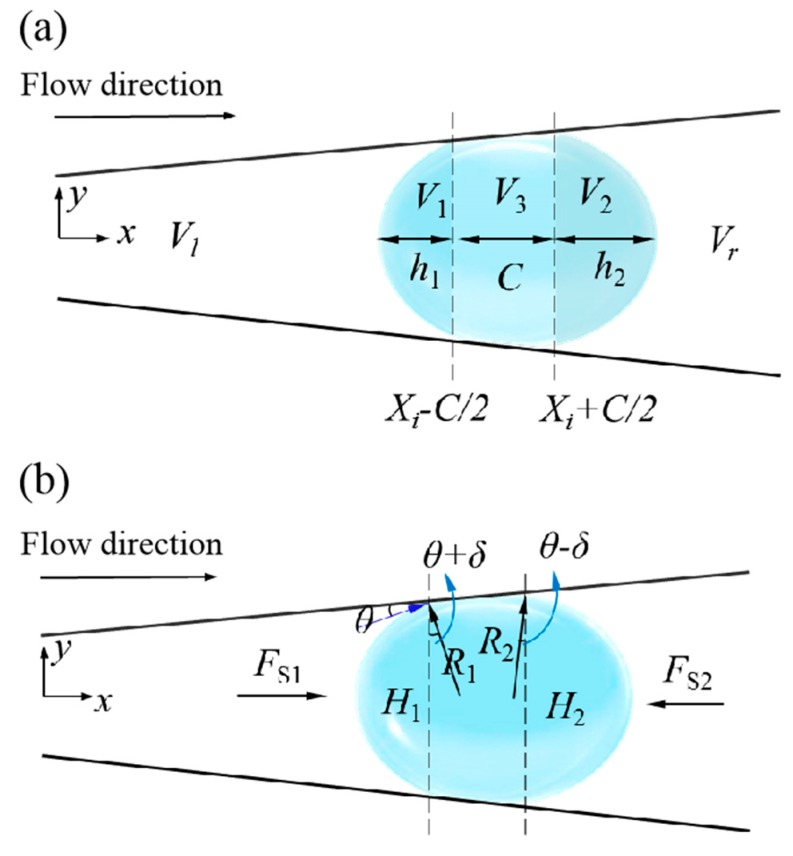
The contact of the bubble with the inner wall of the micromotor. (**a**) The volume of several parts as the bubble is in full contact with the inner wall of the micromotor. (**b**) The geometry of the bubble and the forces it experiences.

As shown in Figure 4a, the volume of the fluid in the tube is divided into two parts. Then the left and right spheres and the volume of the trough are

(29) $$\begin{array}{c} V_{1}=\frac{\pi }{3}h_{1}^{2}(3R_{1}-h_{1})=\frac{\pi }{3}{\left[\frac{(X_{i}-C/2)\tan\delta +R_{\min}}{\cos(\theta +\delta )}\right]}^{3}[2-3\sin(\theta +\delta )+\sin^{3}(\theta +\delta )]\\ V_{2}=\frac{\pi }{3}h_{2}^{2}(3R_{2}-h_{2})=\frac{\pi }{3}{\left[\frac{(X_{i}+C/2)\tan\delta +R_{\min}}{\cos(\theta -\delta )}\right]}^{3}[2-3\sin(\theta -\delta )+\sin^{3}(\theta -\delta )]\\ V_{3}=\frac{\pi }{3}C(H_{1}^{2}/4+H_{1}H_{2}/4+H_{2}^{2}/4)\\ =\frac{\pi }{3}C[R_{1}^{2}\cos^{2}(\theta +\delta )+R_{1}R_{2}\cos(\theta +\delta )\cdot \cos(\theta -\delta )+R_{2}^{2}\cos^{2}(\theta -\delta )]\\ =\frac{\pi }{3}C[R_{1}^{2}\cos^{2}(\theta +\delta )+R_{1}R_{2}(\cos^{2}\theta -\sin^{2}\delta )+R_{2}^{2}\cos^{2}(\theta -\delta )] \end{array}$$

The total volume of the bubble is $V=qt=C_{hd}nSt$, the expression is

(30) $$\begin{array}{c} V=V_{1}+V_{2}+V_{3}\\ =\frac{\pi }{3}R_{1}^{3}[2-3\sin(\theta +\delta )+\sin^{3}(\theta +\delta )]-\frac{\pi }{3}R_{2}^{3}[2-3\sin(\theta -\delta )+\sin^{3}(\theta -\delta )]\\ +\frac{\pi }{3}C[R_{1}^{2}\cos^{2}(\theta +\delta )+R_{1}R_{2}(\cos^{2}\theta -\sin^{2}\delta )+R_{2}^{2}\cos^{2}(\theta -\delta )] \end{array}$$

The left and right fluid volumes are

(31) $$\{\begin{array}{l} V_{l}=\frac{\pi }{3}X_{i}(\frac{R_{\min}^{2}}{4}+\frac{R_{\min}H_{1}}{4}+\frac{H_{1}^{2}}{4})-V_{1}\\ V_{r}=\frac{\pi }{3}(L-X_{i})(\frac{H_{2}^{2}}{4}+\frac{R_{\max}H_{2}}{4}+\frac{R_{\max}^{2}}{4})-V_{2} \end{array}$$

The fluid flow rates at the left and right openings are

(32) $$v_{l}=\frac{1}{A_{1}}\frac{dV_{l}}{dt},\;v_{r}=-\frac{1}{A_{2}}\frac{dV_{r}}{dt}$$

Then the driving force of the tubular micromotor is

(33) $$F_{\mathrm{jet}3}=\rho_{l}\frac{1}{A_{1}}{\left(\frac{dV_{l}}{dt}\right)}^{2}-\rho_{l}\frac{1}{A_{2}}{\left(\frac{dV_{r}}{dt}\right)}^{2}$$

### 2.4. Bubble Ejection

**Figure 5:**
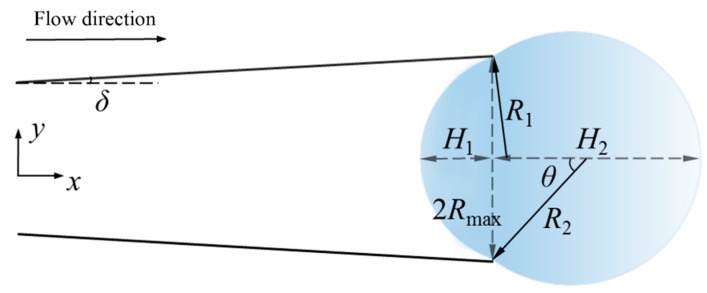
A schematic diagram of the ejection stage of the bubble at the opening of the micromotor.

The asymmetric deformation of the bubble due to buoyancy is not considered at the beginning, as shown in Figure 5.

The radius of the two spheres is *R*_1_ and *R*_2_, respectively, and the volume of the two spheres is *V*_1_ and *V*_2_. $R_{1}=R_{\max}/\cos\delta$, $H_{1}=R_{1}-R_{\max}\tan\delta =R_{\max}/\cos\delta -R_{\max}\tan\delta$

(34) $$V_{1}=\frac{\pi }{3}H_{1}^{2}(3R_{1}-H_{1})=\frac{\pi R_{\max}^{3}}{3\cos^{3}\delta }(\sin^{3}\delta -3\sin\delta +2)$$

and $R_{2}=R_{\max}/\sin\theta$, $H_{2}=R_{2}+R_{\max}/\tan\theta =R_{\max}/\sin\theta +R_{\max}/\tan\theta$

(35) $$V_{2}=\frac{\pi }{3}H_{2}^{2}(3R_{2}-H_{2})=\frac{\pi R_{\max}^{3}}{3\sin^{3}\theta }(-\cos^{3}\theta +3\cos\theta +2)$$

Total bubble volume is

(36) $$V=V_{1}+V_{2}=\frac{\pi R_{\max}^{3}}{3}[\frac{\sin^{3}\delta -3\sin\delta +2}{\cos^{3}\delta }+\frac{-\cos^{3}\theta +3\cos\theta +2}{\sin^{3}\theta }]$$

The bubble moves outward to the opening, where $\theta \in \left(\pi /2,\theta_{t}\right)$, *θ_t_* is the angle at which the bubble is ejected. The main change in bubble volume is reflected in the *V*_2_ part,

(37) $$dV_{2}=-\frac{\pi R_{\max}^{3}{\left(1-\cos\theta \right)}^{2}}{\sin^{4}\theta }d\theta$$

**Figure 6:**
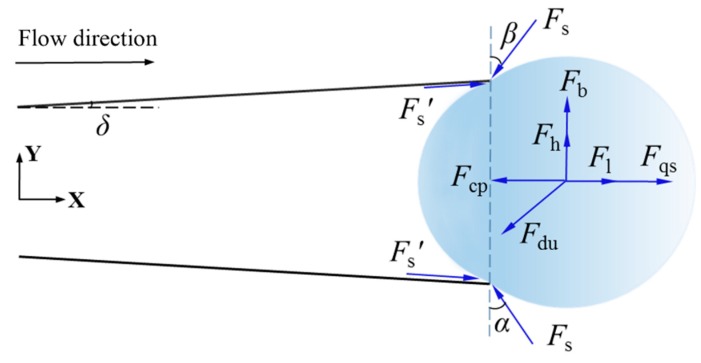
Various forces in the stage of bubble ejection of a conical micromotor.

The forces applied to the stage of bubble ejection are shown in Figure 6.

The surface tension

(38) $$F_{sx}=-F_{s}\cdot \sin\theta +F_{s^{\prime }}\cdot \cos\delta =2\pi \sigma R_{\max}(\cos\delta -\sin\theta )$$

If the bubble growth obeys R = γin, the equivalent bubble radius R′ can be found.

(39) $$F_{qs}=(\frac{2}{3}+\frac{1}{12}Re)\cdot 6\pi \mu u_{s}R^{\prime },\;Re=\frac{\rho_{l}u_{s}2R^{\prime }}{\mu }$$

The unsteady force of asymmetric growth of bubbles is

(40) $$F_{du}=\pi R^{2}\rho_{l}(R\ddot{R}+\frac{3}{2}{\dot{R}}^{2})=-\frac{1}{18}\pi \rho_{l}{\left(\frac{3q}{4\pi }\right)}^{4/3}t^{-2/3}$$

If only considering the buoyancy of the portion of the opening that is exposed to the volume of the *V_2_* portion,

(41) $$F_{b}=(\rho_{l}-\rho_{b})gV_{2}$$

In this stage, the bubble is only in contact with the opening, and the stage radius *d_w_* is substantially close to zero. Consequently, the flow pressure, contact pressure, and shear lift force are zero

(42) $$F_{h}=0,\;F_{cp}=0,\;F_{sl}=0$$

The resultant force of the bubble in the fluid flow direction and the vertical direction is

(43) $$\{\begin{array}{l} F_{x}=F_{s}+F_{qs}+F_{dux}\\ F_{y}=F_{b}+F_{duy} \end{array}$$

According to the flow rate and speed of the openings of the tubular micromotor, the driving force can be obtained

(44) $$F_{\mathrm{jet}4}=\rho_{l}\frac{1}{A_{1}}{\left(\frac{dV}{dt}\right)}^{2}-\rho_{l}\frac{1}{A_{2}}{\left(\frac{dV}{dt}\right)}^{2}$$

During the stage of bubble ejection, the velocity of the fluid flowing through the opening is approximately the same as the velocity of the ejected bubble, which is,

(45) $$\frac{dV}{dt}=\pi R_{\max}^{2}v_{b}$$

where *v_b_* is the speed at which the bubble is ejected from the opening.

Assuming that the bubble and the micromotor wall are smooth and frictionless, the work done due to the difference in surface tension on the bubble is all converted into the kinetic energy of the bubble, namely

(46) $$\frac{1}{2}m_{b}v_{b}^{2}=W_{S1}-W_{S2}$$

The bubble is ejected from the opening at a speed of *v_b_*

(47) $$\{\begin{array}{l} W_{S1}=\int_{0}^{L-X_{i}+C/2}F_{S1}dx=\pi \sigma \cos(\theta +\delta )[{\left(L-X_{i}\right)}^{2}\tan\delta +2R_{\min}(L-X_{i}+C/2)]\\ W_{S2}=\int_{0}^{L-X_{i}-C/2}F_{S2}dx=\pi \sigma \cos(\theta -\delta )[{\left(L-X_{i}\right)}^{2}\tan\delta +2R_{\min}(L-X_{i}-C/2)] \end{array}$$

The speed when the bubble is ejected is

(48) $$v_{b}=\frac{2\pi \sigma }{\rho_{g}V_{g}}{\left\{\cos\theta \cos\delta R_{\min}C-2\sin\theta \sin\delta [{\left(L-X_{i}\right)}^{2}\tan\delta +2R_{\min}(L-X_{i})]\right\}}^{\frac{1}{2}}$$

Then driving force during the stage could be expressed as

(49) $$\begin{array}{ll} F_{\mathrm{jet}4}= & \frac{4\pi^{3}\sigma^{2}\rho_{l}R_{\max}^{2}(R_{\max}^{2}-R_{\min}^{2})}{\rho_{g}^{2}V_{g}^{2}R_{\min}^{2}}\{\cos\theta \cos\delta R_{\min}C-\\ & 2\sin\theta \sin\delta [{\left(L-X_{i}\right)}^{2}\tan\delta +2R_{\min}(L-X_{i})]\} \end{array}$$

### 2.5. Micromotor Velocity 

Here we show a simplified version of the micromotor motion theory based on driving force and drag force. A detailed theoretical study of drag force can be found in Wang et al. [23]. According to Newton’s second law, the movement of a tubular micromotor immersed in a fluid can be expressed as

(50) $$m\dot{v}=\Sigma F=F_{\mathrm{jet}}-F_{d}$$

where *F_d_* is the drag force experienced by the micromotor. The relationship between *F_d_* and Reynolds number, micromotor geometry, and the drag coefficient is obtained by the computational fluid dynamics software FLUENT 18.0 (ANSYS).

The mass of the micromotor *m* is

(51) $$\begin{array}{ll} m=\rho V_{j}= & \rho [\frac{\pi }{3}L\left({R_{\max}}^{2}+R_{\max}R_{\min}+{R_{\min}}^{2}\right)\\ & \;-\frac{\pi }{3}L\left({R_{\max}}^{\prime 2}+{R_{\max}}^{\prime }{R_{\min}}^{\prime }+{R_{\min}}^{\prime 2}\right)] \end{array}$$

The drag force is [23]

(52) $$F_{d}=\frac{\pi }{4}\mu vR_{\max}\left[\left(ae^{b\lambda }+\frac{c}{\xi }+\frac{d}{\xi^{2}}\right)\tan\delta +\frac{f}{\xi }\right]$$

Solving the differential equation of Equation (51), the expression of the motion velocity of the tubular micromotor could be obtained on the basis of driving force and drag force.

## 3. Results and Discussion

### 3.1. Bubble Nucleation and Growth 

**Figure 7:**
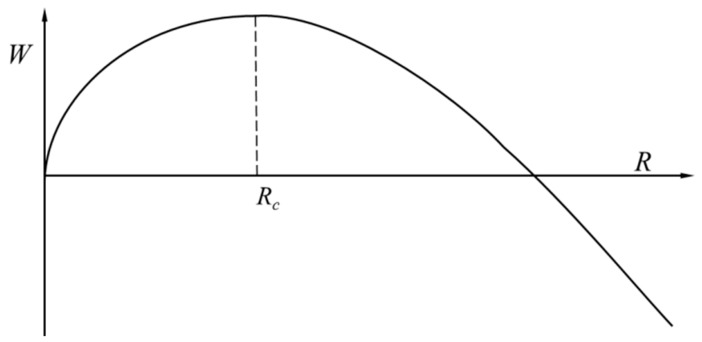
Relationship between nucleation free energy change and the nucleus radius of the bubble.

The relationship between the nucleation free energy change W and the nucleus radius R is shown in Figure 7.

When the bubble nucleates on the inner wall of the micromotor, the volume fraction of fluid discharged from the left end is *f* = 0.358. The computation is fulfilled by FLUENT. The relationship between the moving speed of the tubular micromotor and the solution surface tension coefficient, the viscosity, concentration, motor tube length, opening radius, and the semi-cone angle were calculated.

**Figure 8:**
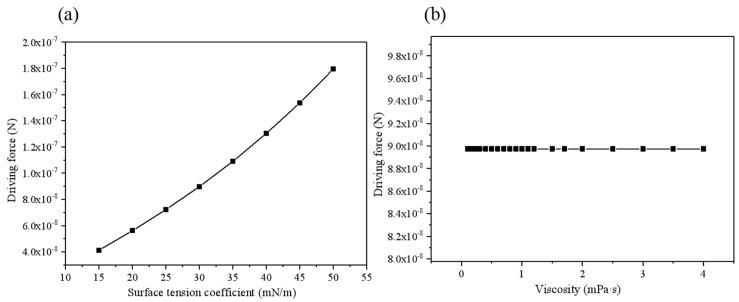
Relationship between driving force and (**a**) surface tension coefficient (**b**) fluid viscosity.

**Figure 9:**
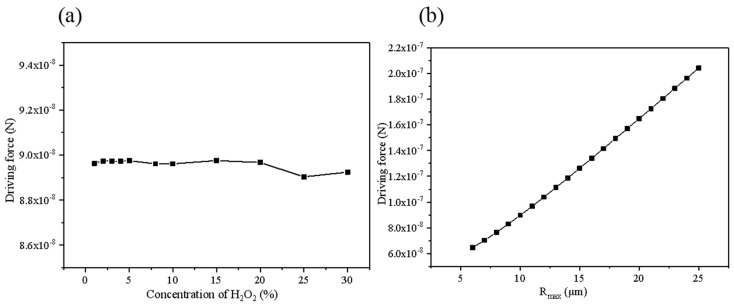
Relationship between driving force and (**a**) concentration of hydrogen peroxide C_H2O2_ and (**b**) opening radius.

**Figure 10:**
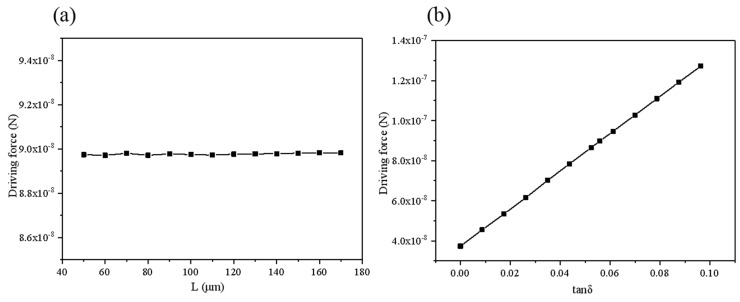
Relationship between driving force and (**a**) micromotor length (**b**) tangent semi-cone angle.

The data in Figure 8 is fitted to obtain the relationship between the driving force and the surface tension coefficient, *F*_jet2_ = *f* (*σ*^2^). As can be seen from Figure 8b, when the viscosity changes from 0.1 mPa·s to 4.0 mPa·s, the driving force remains substantially unchanged. Thus, the fluid viscosity almost has no influence on the driving force in the stage. When the concentration of the hydrogen peroxide solution in Figure 9a changes, the speed does not change much, and the difference between the maximum and minimum values is only 0.62%. Basically, it can be considered that the change in the concentration of the peroxide solution almost has no effect on the driving force. Fitting the curve in Figure 9b to obtain the relationship between the driving force and the opening radius $F_{\mathrm{jet}2}=f(R_{\max}^{3})$.The relationship with the micromotor length and semi-cone angle is linear, as demonstrated by Figure 10a,b. θ is the contact angle of the bubble with the micromotor wall, and *φ* is the inclination angle of the bubble under the action of the flow rate. The front and back contact angle *α* and *β* of the surface tension in Equation (18) are determined by both contact angels. Furthermore, Equation (19) used for calculation of the bubble radius R is related to the contact angle *θ*. Considering Equations (20)–(27) are all related to the bubble radius R, the formula of the driving force could be expressed as a function of *θ* and *φ*, namely *f*(*θ*) and *g*(*φ*).

Combing the relationship between *F*_jet2_ and *σ* as well as *R*_max_, the final expression of the driving force in the stage is

(53) $$F_{\mathrm{jet}2}=\chi \rho_{l}\sigma^{2}R_{\max}^{3}\tan\delta f(\theta )g(\varphi )$$

where χ is a conversion coefficient, which means that the resultant force of the fluid and solid motor acting on the bubble along the direction of the fluid flow cannot be completely converted to the driving force of the micromotor. The conversion coefficient χ can be obtained from experimental results in the literature [20]. It should be noted that Equation (53) is only used to characterize the relationship between the driving force and other parameters. In fact, the driving force is obtained from Equations (16) and (28). 

### 3.2. Driving Force of the Full Life-Cycle of the Bubbles

**Figure 11:**
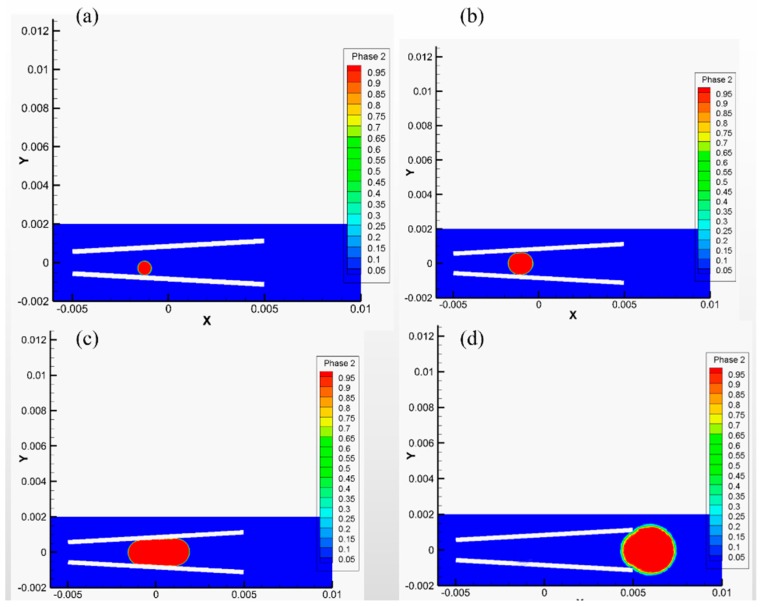
A phase diagram of bubbles in the stage of (**a**) bubble nucleation (**b**) bubble growth (**c**) bubble slip (**d**) bubble ejection, where red represents the gas and blue represents the fluid.

Taking a peroxide solution with a concentration of 5% as an example, the density is *ρ_l_* = 10^3^ kg/m^3^, the viscosity *μ* = 0.9 mPa⋅s, the surface tension coefficient *σ* = 30 mN/m. The geometry of the tubular micromotor has: length *L* = 100 μm, larger opening radius *R*_max_ = 10 μm and semi-cone angle *δ* = 3.2°. The driving forces at each stage are shown in Table 1. A phase diagram of the bubble in the full life cycle of bubbles can be found in Figure 11.

As can be seen from Table 1, the bubbles contribute to the movement of the tubular micromotor at various stages of micromotor motion. The driving forces contributed by bubble nucleation and slip are insignificant. It is worth mentioning that, the driving force contributed by the nucleation stage is almost negligible. The driving forces contributed by bubble growth and ejection are prominent, as can be seen from Table 1. Bubbles provide most of the driving force during the stage of growth and ejection. Our results are a quantitative comparison of the driving forces generated by bubbles in the four stages of the full life cycle of bubbles, which is more comprehensive than the previous reports on the individual stages of the research [17,18,19]. The four stages of the bubble life cycle are strictly distinguished and compared quantificationally separately. A similar conclusion provided by ref. [12] is from experimental observation and is only a qualitative conclusion while our results further provide a quantitative description to arrive at the conclusion that the driving force is mainly from the growth stage. For example, the driving force of the growth stage accounts for the ratio of the total driving force. The results also confirmed results reported by Fomin et al. that both bubble growth and ejection generate the driving force [20]. Furthermore, our results used detailed computations to reveal that bubble growth and ejection provide the primary driving force for micromotors. 

### 3.3. The Influence of Driving Force on Speed in Each Stage

**Figure 12:**
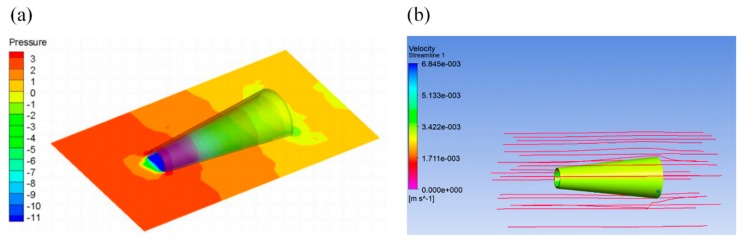
Tubular micromotor (**a**) pressure distribution and (**b**) streamline.

Taking the Reynolds number Re = 0.0125 in Table 2 as an example, FLUENT. calculates the pressure distribution cloud diagram and streamline diagram on the tubular motor as shown in Figure 12.

The results show that the pressure near the upstream ring is larger and the pressure at the tail of the motor tube becomes smaller. The drag forces of all the walls of the tubular micromotor were monitored separately, including the upstream ring, the backflow ring, the outer wall, and the inner wall. Drag forces of various areas are shown in Table 2.

The results demonstrated that the drag forces of the outer wall were the smallest. The drag forces on the region of the upstream and backflow ring were small, and the drag forces on the inner wall were the largest. Therefore, the drag forces of the tubular motor mainly come from the inner wall of the micromotor body. Considering the drag forces of the inner wall of the tube, we obtained a more accurate drag force for conical tubular micromotors

(54) $$F_{d}=\frac{\pi }{4}\mu vR_{\max}\left[\left(ae^{b\lambda }+\frac{c}{\xi }+\frac{d}{\xi^{2}}\right)\tan\delta +\frac{f}{\xi }\right]$$

**Figure 13:**
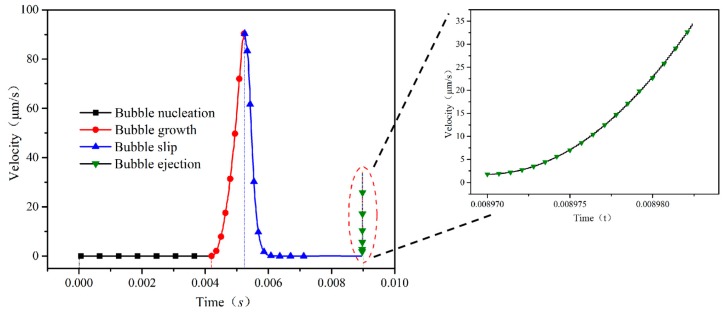
The velocity of the micromotor in the full life-cycles of bubbles, namely bubble nucleation, growth, slip, and ejection. Inset: ejection stage.

The effect of the driving force generated by the bubble at each stage on the motor speed was evaluated. The dimensions and fluid parameters for a given tubular micromotor are as follows: 5% hydrogen peroxide solution, density *ρ_l_* = 1130 kg/m^3^, viscosity *μ* = 0.9 mPa⋅s, surface tension coefficient *σ* = 30 mN/m; micromotor length *L* = 100 μm, larger opening radius *R*_max_ = 10 μm, semi-cone angle *δ* = 3.2°; solution density *ρ_g_* = 1.33, contact angle *α* = 45°, *β* = 36°, angle of inclination *φ* = 4.5°. The viscosity *μ* = 0.9 mPa⋅s that we used falls into the general viscosity of the fluid ranges from 0.1–4 mPa·s. The generation rate of bubbles in the chemical reaction is *n* = 9.8 × 10^−4^ m/s. For example, when the random nucleation point is in the middle of the tube, the stage of bubble ejection has a shorter duration. The velocity of the tubular micromotor in the various stages of the bubble is as shown in Figure 13.

As can be seen in Figure 13, the nucleation stage takes the longest time and the ejection stage takes the shortest time during the entire bubble cycle. The stage of bubble nucleation generates driving forces but has a limited contribution to the motion. The growth stage plays a major role in the micromotor motion, as seen in Figure 13. The speed of the motor rapidly increases in the stage. However, the driving force in the slip stage drops rapidly and the speed of the micromotor is accelerated to a higher level than the previous stage. The drag forces in the slip stage are also larger, even exceeding the driving force temporally. Therefore, the micromotor exhibits a decrease in speed in the stage. On the contrary, a decrease in the speed reduces fluid resistance, causing the speed to decrease more and more slowly. The bubble finally approaches an equilibrium state until it leaves the opening and bursts [18].

It can be seen from Figure 13, the velocity of the bubble in the ejection stage is increased, but the increase is smaller than the growth stage. Although the driving force in the ejection stage is large, the duration of the stage is too short to make a comparable contribution to the micromotor motion. Among the four stages of the bubble life cycle, the most prominent important contribution to micromotor motion is the growth stage, as seen in Figure 13 and the most insignificant contribution to micromotor motion is the nucleation stage. The contribution of the whole bubble cycle to the velocity of the tubular micromotor is averaged by integrating the velocity of the whole life-cycle of the bubble using the formula. The calculations show that the average velocity is about 6.49 μm/s when the bubbles nucleate in the middle of the micromotor. 

Bubbles are produced in large quantities at a certain frequency to propel the micromotor ahead [35]. A further consideration is given to the effect of the generation of numerous bubbles on the motor motion. The frequency of generated bubbles used in the analysis was the same as that of Li et al. [5]. When the semi-cone angle of the tubular micromotor increased from 0° to 5°, the frequency of bubbles decreased from 97 Hz to 54 Hz which can be obtained by linear interpolation. When the semi-cone angle is 3.2°, the bubble generation frequency should be 69.48 Hz. Micromotor geometry used in the analysis has length *L* = 100 μm, the larger opening radius *R*_max_ = 10 μm, semi-cone angle *δ* = 3.2°. When moving in 5% peroxide solution, the average speed of motion is 450.93 μm/s which is in accordance with 431 μm/s reported by Li et al [5].

**Figure 14:**
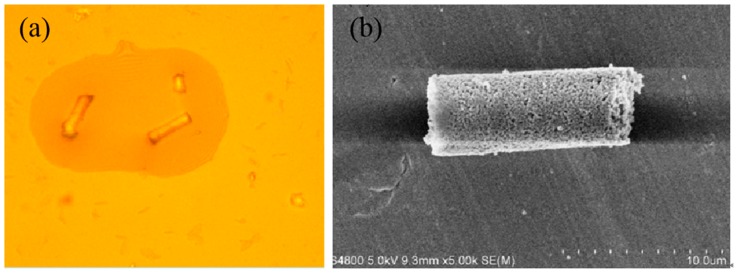
Optical microscopy and scanning electron microscopy of tubular micromotor.

We further validated our theory of driving forces with experimental measurements. The sample was placed in an aqueous solution of hydrogen peroxide, and the speed of the micromotor was measured to compare with the theoretical results. Figure 14 shows the images of optical microscopy and scanning electron microscopy of the tubular micromotor prepared by ourselves. The parameters for the micromotor are as follows: 5% hydrogen peroxide solution, density *ρ_l_* = 1130 kg/m^3^, viscosity *μ* = 0.9 mPa⋅s, surface tension coefficient *σ* = 30 mN/m, micromotor length *L* = 100 μm, larger opening radius *R*_max_ = 10 μm, semi-cone angle *δ* = 3.2°, solution density *ρ_g_* = 1.33, contact angle *α* = 45°, *β* = 36°, angle of inclination *φ* = 4.5°.

**Figure 15:**
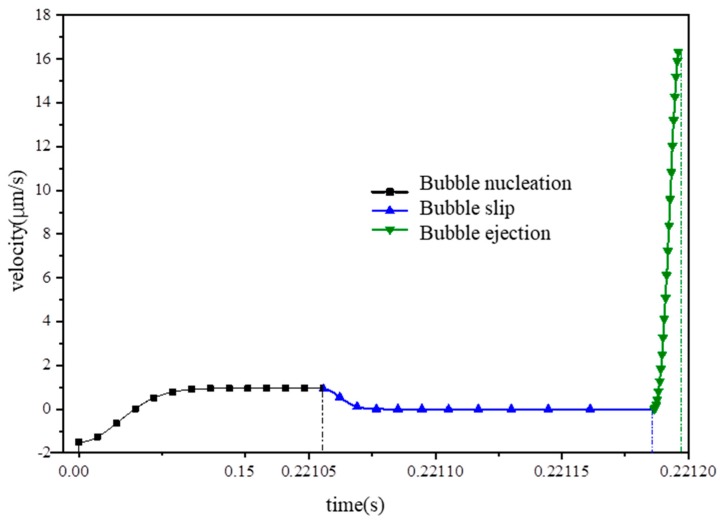
The velocity of the tubular micromotor in the stage of bubble nucleation, slip, and ejection.

Because the micromotor used in the experiment has a small size, the bubbles quickly contact the wall after nucleation and then go to the slip stage. In this case, the life-cycle of the bubbles appears as three stages in the absence of the growth stage. The velocity of the micromotor in the life-cycle can be found in Figure 15. The average velocity of the micromotor in a single bubble cycle computed by our theory is 7.39 μm/s. The experimentally measured frequency of bubbles is 75 Hz. The average speed obtained from theory is 554.25 μm/s. Moreover, the average speed obtained from the measured trajectory is 540 μm/s. Consequently, the experimental results agree very well with the theoretical prediction.

## 4. Conclusions

The dynamical behavior of the bubble plays a crucial role throughout the movement of the bubble-driven tubular micromotor. In this paper, based on the unclear problem of the driving mechanism of the bubble-driven micromotor, the theoretical model of the driving force of the motor in different stages was established by using mass transfer model, hydrodynamic theory, and numerical simulation. Based on the bubble nucleation theory, a theoretical calculation model of the driving force of the tubular micromotor in the nucleation stage of the bubble was determined. Based on the principle of force balance, the theoretical calculation model of the driving force of the tubular micromotor in the growth stage of the bubble tube was established. Based on the mass transfer theory, a theoretical model of the driving force of the motor in the slip stage of the bubble tube was derived. Based on the principle of force and energy conservation, a theoretical model of the driving force of the tubular micromotor in the ejection stage of the bubble was formed. The results show that the driving forces produced by bubble nucleation and the slip are insignificant. The driving force contributed by the bubble nucleation is the smallest and is almost negligible. The stage of bubble growth and ejection provide most of the driving force. Further, comparing the contribution of the driving force at each stage to the motor speed, it was found that the growth stage plays a major role in the locomotion of the bubble-driven micromotor. The micromotor speed based on the driving force of the bubble life-cycle is in consistent with the experimental results.

## Figures and Tables

**Table 1:** The driving forces, average speed, and duration of the bubble-driven micromotor at different stages.

Stages	Equation	Driving Force	Average Speed (μm/s)	Duration (ms)
Nucleation	(14)	0.03958	6.808 × 10^−4^	0–4.2
Growth	(56)	2995	1011	4.2–5.25
Slip	(33)	18.83	191.4	5.25–8.97
Ejection	(49)	2360	15.65	8.97–8.98

**Table 2:** Drag forces of the various surfaces of the tubular motor.

Surface Area	Upstream Ring	Backflow Ring	Outer Wall	Inner Wall
Drag force (nN)	0.028	0.043	0.002	0.191
Drag force (nN)	0.264			
